# Relationship between Ventilator-Associated Events and Timing of Rehabilitation in Subjects with Emergency Tracheal Intubation at Early Mobilization Facility

**DOI:** 10.3390/ijerph15122892

**Published:** 2018-12-17

**Authors:** Taku Shinoda, Hiromasa Nishihara, Takayuki Shimogai, Tsubasa Ito, Ryuya Takimoto, Ryutaro Seo, Masashi Kanai, Kazuhiro P. Izawa, Kentaro Iwata

**Affiliations:** 1 Department of Rehabilitation, Division of Physical Therapy, Kobe City Medical Center General Hospital, Kobe 650-0047, Japan; t135x246s88@yahoo.co.jp (T.S.); ardssepsis@gmail.com (H.N.); tak813hir@gmail.com (T.S.); tsubasa_ito_wally@yahoo.co.jp (T.I.); taki_ku_19@yahoo.co.jp (R.T.); iwaken@kcho.jp (K.I.); 2 Department of Public Health, Kobe University Graduate School of Health Sciences, Kobe 654-0142, Japan; kanaimasa07@gamail.com; 3 Cardiovascular Stroke Renal Project (CRP), Kobe 654-0142, Japan; 4 Department of Emergency Medicine, Kobe City Medical Center General Hospital, Kobe 650-0047, Japan; ryutaro_seo@hotmail.com

**Keywords:** ventilator-associated events, timing of rehabilitation, early mobilization, intubated trachea, retrospective research, rehabilitation protocol

## Abstract

The present study aimed to investigate the relationship between the occurrence of ventilator-associated events (VAE) in the intensive care unit and the timing of rehabilitation intervention. We included subjects who underwent emergency tracheal intubation and received rehabilitation. We performed rehabilitation according to our hospital’s protocol. We assessed the mechanical ventilation parameters of inspired oxygen fraction and positive-end expiratory pressure, and a VAE was identified if these parameters stabilized or decreased for ≥2 days and then had to be increased for ≥2 days. We defined time in hours from tracheal intubation to the first rehabilitation intervention as Timing 1 and that to first sitting on the edge of the bed as Timing 2. Data were analyzed by the *t*-test and χ^2^ tests. We finally analyzed 294 subjects. VAE occurred in 9.9% and high mortality at 48.3%. Median values of Timing 1 and Timing 2 in the non-VAE and VAE groups were 30.3 ± 24.0 and 30.0 ± 20.7 h, and 125.7 ± 136.6 and 127.9 ± 111.4 h, respectively, and the differences were not significant (*p* = 0.95 and *p* = 0.93, respectively). We found no significant relationship between the occurrence of VAE leading to high mortality and timing of rehabilitation intervention.

## 1. Introduction

After admission to the intensive care unit (ICU), patients are in danger of complications such as sepsis, ICU-acquired weakness, and delirium [1,2,3]. In particular, pneumonia is a common complication in critically ill ICU patients on mechanical ventilation. Ventilator-associated pneumonia (VAP) prolongs the length of stay (LOS) in hospital and increases mortality. Mortality rates of 24 to 50% have been reported because of VAP [4,5]. Therefore, we need to explore means to prevent VAP.

However, VAP is problematic because it lacks objective and gold-standard criteria, thus is difficult to diagnose. Therefore, the Center for Disease Control and Prevention (CDC) established a new surveillance definition termed ventilator-associated events (VAE). Although VAE is not used in clinical situations, the definition has been retrospectively used for the objective surveillance of medical records [6,7,8].

VAE is identified according to the following factors of mechanical ventilation. As prerequisites, the fraction of inspiratory oxygen (FiO_2_) and positive end-expiratory pressure (PEEP) need to be stabilized or decreased for a period of ≥2 calendar days (defined as the patient being on mechanical ventilation from one day to the next). After that, the FiO_2_ or PEEP is required to have been increased for a period of ≥2 calendar days because of clinical events. Thus, the diagnosis of VAE requires at least four full days to meet the definition.

Clinical events that can cause the above ventilator changes include not only pneumonia, but also conditions such as acute respiratory distress syndrome, atelectasis, and pulmonary edema according to the CDC National Healthcare Safety Network [9]. Although these are common clinical conditions in the severely ill, they may be preventable [10].

Several similar studies have reported that early mobilization decreases the onset of VAP [11,12]. For example, Titsworth et al. reported that progressive upright mobility significantly decreased the incidence of VAP [13]. For the prevention of VAE, however, although potential strategies including early mobilization and mobility were discussed, they were not actually considered to be feasible [10]. Furthermore, a clinical review in 2013 reported that early mobilization was variously defined to mean within two to five days of the onset of critical illness [14]. However, in another report, early rehabilitation was defined as methods of support provided for a patient within 48 hours from disease onset, surgery, or acute exacerbation [15]. Nevertheless, no reports have that defined “early” in terms of a specific and optimal time [16].

We hypothesized that the earlier rehabilitation intervention could be performed, the more easily VAE could be prevented. Therefore, the aim of this study was to investigate the relationship between the occurrence of VAE during hospitalization and timing from tracheal intubation to rehabilitation intervention, and to elucidate the disease background of the present study subjects diagnosed as having VAE.

## 2. Materials and Methods

### 2.1. Study Design

This retrospective single-facility study was performed at Kobe City Medical Center General Hospital, Hyogo Prefecture, Japan. Our hospital has eight beds in the emergency ICU and six beds in the coronary care unit.

### 2.2. Study Subjects

We included 851 consecutive subjects who were admitted from April 2014 to May 2017, and who were managed by the emergency department, underwent emergent tracheal intubation for the first time, were >18 years old, and received rehabilitation. We excluded subjects who required mechanical ventilator support for ≤3 calendar days [8] or had missing data. As we analyzed the time to sitting on the edge of the bed, we also excluded subjects whose condition was judged to be too poor to undergo the rehabilitation protocol and who could not sit on the edge of the bed.

### 2.3. Rehabilitation

**Figure 1:**
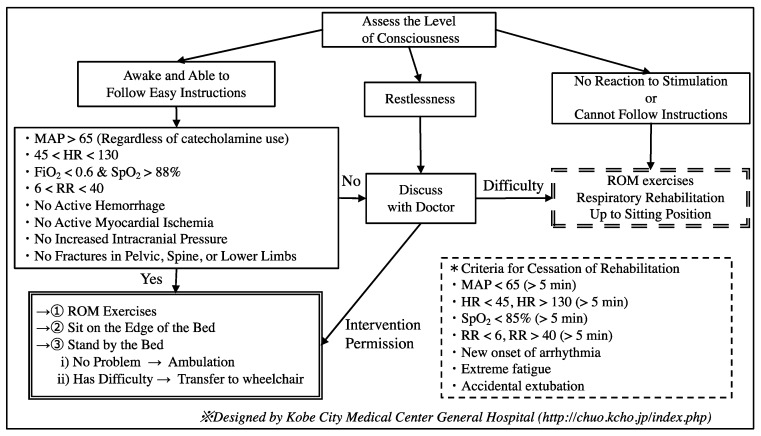
The rehabilitation protocol used in our hospital. (1) We first assessed the level of consciousness. If the patient was awake and could follow easy instructions, we assessed their vital signs and physical findings. If satisfactory, we performed rehabilitation exercises out of bed as shown in the double-line box. Otherwise, we discussed with the physician whether it was possible to perform rehabilitation exercises. (2) If the patient showed restlessness, we also discussed this same issue with the physician. (3) If the patient did not react to stimuli and could not follow easy instructions, we performed rehabilitation on the bed as shown in the double-dashed-line box. If the patient met any of the criteria in the dashed-line box, we ceased rehabilitation. MAP = mean arterial pressure, HR = heart rate, FiO_2_ = fraction of inspiratory oxygen, RR = respiratory rate, and ROM = range of motion.

In principle, we used the rehabilitation protocol designed by the Kobe Medical Center General Hospital Emergency and Anesthesiology Department from 2014, as shown in Figure 1. 

In our hospital, a physician orders rehabilitation as soon as the patient enters the ICU. Unless in the middle of the night or the patient’s unstable condition requires urgent action, we will intervene with rehabilitation as soon as possible. Furthermore, in our hospital, we share information on the patient’s condition, the previous day’s rehabilitation situation, and the rehabilitation program for the day between the rehabilitation staff (physical and occupational therapists), physicians, and nurses every morning. Each day, we adjust the intervention time and share information again with the nurse just before the intervention. We intervened with help from the nurse from 9:30–16:30 on Monday to Friday and from 9:30–12:00 on Saturday and Sunday.

We defined two types of rehabilitation timing in units of hours: the time from tracheal intubation to the first rehabilitation intervention (regardless of its contents) was defined as Timing 1, and the time from tracheal intubation to first sitting on the edge of the bed (regardless of assistance) was defined as Timing 2.

### 2.4. Data Collection

The following data were collected from the subjects’ computerized medical records: age, body mass index (BMI), sex, principal diagnostic reason for tracheal intubation, Acute Physiology and Chronic Health Evaluation II (APACHE II) score [17], LOS during hospitalization, hospital mortality, and rates of weaning from the ventilator (including death with intubation), experience of re-intubation, and tracheostomy.

We collected the daily values of minimum FiO_2_ and PEEP to define VAE. There are two sets of criteria for VAE as defined by the CDC National Healthcare Safety Network [9]: (1) the daily minimum FiO_2_ should be stable or decreasing for at least two calendar days, followed by an increase of at least 0.20 and maintained at that level for at least two calendar days, and (2) the daily minimum PEEP should be stable or decreasing for at least two calendar days, followed by an increase of at least 3 cm H_2_O and maintained at that level for at least two calendar days. We diagnosed VAE according to the presence of either condition. We investigated the clinical events that caused the VAE. As we counted all of the events that we considered to be candidates, there was some overlap.

### 2.5. Statistical Analysis

We divided the subjects into two groups according to the occurrence of VAE: the non-VAE group and VAE group. All quantitative variables were expressed as the mean ± standard deviation, and all qualitative variables were expressed as the number and percentage of the group. Quantitative variables were compared by the unpaired Student *t*-test, and qualitative variables were compared by the χ^2^ test between the two groups. A *p* value of <0.05 was considered to indicate statistical significance. Statistical analyses were performed with IBM SPSS 24.0 J statistical software (IBM SPSS Japan, Inc., Tokyo, Japan).

### 2.6. Ethics Approval and Consent to Participate

The present study complied with the principles of the Declaration of Helsinki regarding investigations in humans and was approved by the local institutional review board of our hospital (approval no. 180633).

## 3. Results

**Figure 2:**
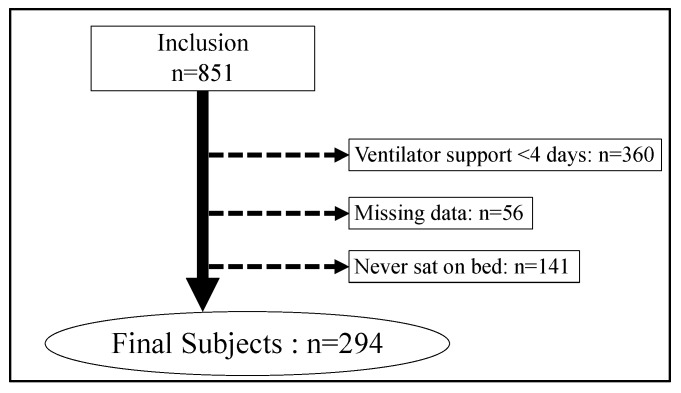
Patient flow in this study.

After applying the exclusion criteria to the 851 subjects, we finally analyzed 294 subjects (Figure 2). Diagnostic reasons for tracheal intubation are shown in Table 1, with the top three reasons being stroke, trauma, and pneumonia.

There were 29 (9.9%) subjects with a VAE. Table 2 shows the characteristics and outcomes of the two patient groups. There were no significant differences between the two groups in age, BMI, sex, APACHE II score, LOS during hospitalization, and rate of tracheostomy. However, there were significant differences between the non-VAE group and VAE group in the rates of hospital mortality (14.0% vs. 48.3%, *p* < 0.001), re-intubation (13.2% vs. 31.0%, *p* = 0.01), and weaning mechanical ventilator (84.2% vs. 44.8%, *p* < 0.001). All patients who died in the VAE group were difficult to wean from the ventilator.

**Figure 3:**
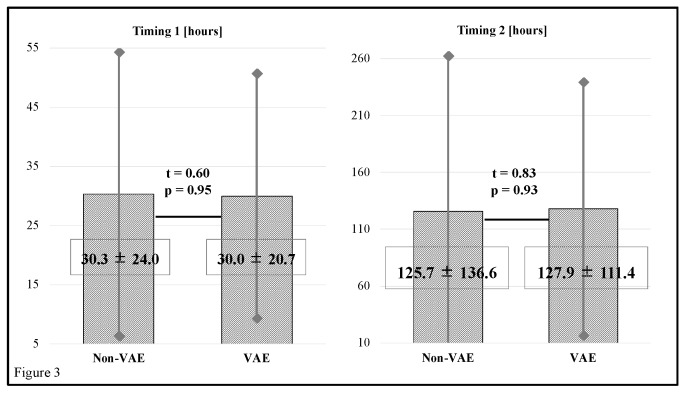
Timing 1 values in the non-VAE group and VAE group were 30.3 ± 24.0 and 30.0 ± 20.7 h, and those of Timing 2 were 125.7 ± 136.6 and 127.9 ± 111.4 h, respectively. The differences were not significant (*t* = 0.60, *p* = 0.95 and *t* = 0.83, *p* = 0.93, respectively). VAE = ventilator-associated events.

Figure 3 shows that there were no significant differences in timing between the non-VAE group and VAE group (*t* = 0.60, *p* = 0.95 and *t* = 0.83, *p* = 0.93, respectively). The respective mean values of Timing 1 in the non-VAE group and VAE group were 30.3 ± 24.0 and 30.0 ± 20.7 h, and those of Timing 2 were 125.7 ± 136.6 and 127.9 ± 111.4 h, respectively. Diagnostic reasons for tracheal intubation in the VAE group subjects are shown in Table 3.

The top four reasons were pneumonia, followed by stroke, trauma, and infection. The top three clinical events causing VAE were pneumonia in 41.4%, pulmonary edema in 34.5%, and atelectasis in 27.6% of the subjects. Some of these clinical events overlapped.

## 4. Discussion

### 4.1. Key Findings

To our knowledge, there have been no reports investigating the relationship between VAE and rehabilitation. Therefore, we investigated the relationship between the occurrence of VAE and the timing of rehabilitation intervention.

Among the 9.9% of subjects in whom a VAE occurred, there was a significantly higher rate of re-intubations and lower probability of weaning from the mechanical ventilator. Moreover, mortality in these subjects was increased when compared with the subjects of the non-VAE group. However, there was no significant difference in the timing of rehabilitation intervention between the two groups.

### 4.2. Study Subjects

We included subjects ventilated for ≥4 calendar days in the present study. Other previous studies used various inclusion criteria such as ≥2 or three calendar days [18,19,20]. Therefore, various rates of occurrence of VAE have been reported. In Japanese hospital data from Kobayashi et al., the included subjects were ventilated for ≥4 calendar days, and the reported rate of occurrence of VAE was approximately 13.4% [21], whereas that of the present study was 9.9%. This may be because we excluded subjects who had never received rehabilitation or had never sat on the edge of the bed. As their severe condition precluded rehabilitation interventions, it is possible that they could also have experienced a VAE.

Consistently high mortality and prolonged mechanical ventilator use due to VAE have been reported in previous studies [19,21]. We also found it difficult to wean the subjects with a VAE from mechanical ventilation, and these subjects experienced high rates of re-intubation and mortality. Thus, the present results supported those of these previous studies. VAE results in a poor prognosis and high mortality, and clearly, it is best if it can be prevented in the first place.

### 4.3. Rehabilitation

In the present study, the first intervention (Timing 1) occurred within approximately 24 h and the first time that the subjects sat on the edge of the bed (Timing 2) occurred within approximately 96 h. In previous definitions, early mobilization was recommended to start within 2–5 days or within 48 h [14,15]. Mendez-Tellez et al. reported that the subjects in their early physical therapy group were out of bed within five days [22], and Morris EP et al. reported that the subjects in their early protocol group were out of bed within 8.5 days [23]. Therefore, we considered that the rehabilitation interventions were performed sufficiently early in the present study.

### 4.4. Relationship between VAE and Rehabilitation Timing

We found no relationship between VAE and rehabilitation timing. The reasons for this may be as follows. First, we had already intervened in all subjects with sufficient early rehabilitation initiated within two to five days of the onset of illness. Thus, it is highly possible that the initiation of rehabilitation had no effect on VAE.

Second, in addition to early rehabilitation, we may require other indices of rehabilitation such as the amount performed, the frequency at which it is performed, and other variables. Yagi et al. reported that early and intensive rehabilitation was important for subjects with stroke [24]. Additionally, alveolar recruitment strategies or lateral-horizontal patient position may reduce complications [25,26]; however, neither has ever been reported to be related with VAE.

Third, the disease background in the study subjects was varied. As nursing care and treatment are different according to disease, progress in rehabilitation may also be different. Moreover, various clinical events caused the VAE. The most frequently reported clinical events associated with VAE were pneumonia or aspiration in first place; pulmonary edema, pleural effusion, or heart failure in second place; and atelectasis in third place [11]. The present study results were similar to those of this previous study. Thus, the preventive strategies used may also need to be different, and we need to focus on the details of these differences.

### 4.5. Limitations

The present study was a single-center, retrospective study. The data gathered on timing probably had a 1–2-h error. Moreover, there were data that we could not track such as comorbidities, medical history, and activities of living before hospitalization. We did not consider missing data in the analysis. It may be necessary to consider other characteristics in the analysis. We also did not consider other rehabilitation parameters (e.g., amount and frequency of rehabilitation), and, in particular, changes in posture. It is necessary to investigate how not only physical therapists but also nurses are involved with patients. Although the diagnosis of VAP is made subjectively, we had to collect data on VAE, which is diagnosed objectively; however, VAE is not used clinically, and the events causing it are diverse.

## 5. Conclusions

We investigated the relationship between the occurrence of VAE and the time from emergency tracheal intubation to rehabilitation intervention in subjects on mechanical ventilation for ≥4 calendar days. VAE occurred in 9.9% of the subjects and caused a poor prognosis and high mortality (48.3%), similar to that reported in previous studies.

The first rehabilitation intervention was performed at a median time of approximately 24 h after tracheal intubation, and the subjects first sat on the edge of the bed within approximately 96 h. However, there was no significant relationship between the occurrence of VAE and the timing of rehabilitation intervention. The clinical events causing VAE were varied, with the leading causes being pneumonia, pulmonary edema, and atelectasis. In addition to the early timing of rehabilitation interventions, intensive rehabilitation and ensuring longer times out of bed may also be needed to prevent VAE.

## Figures and Tables

**Table 1:** Principal diagnostic reasons for tracheal intubation in the study subjects.

Principal Diagnostic Reason	*N* (%)
Stroke	109 (37.1)
Trauma	47 (16.0)
Pneumonia	31 (10.5)
Infection	25 (8.5)
Convulsion	20 (6.8)
Alveolar hemorrhage	7 (2.4)
Hemorrhagic shock	7 (2.4)
Upper airway obstruction	6 (2.0)
Others	42 (14.3)
Total, *n* (%)	294 (100)

**Table 2:** Characteristics and outcomes of the non-VAE and VAE groups.

Clinical Characteristics	Non-VAE	VAE	*t* or χ^2^ Value	*p* Value
	265 (90.1%)	29 (9.9%)		
Age, years, (M ± SD)	67.5 ± 15.8	66.8 ± 15.0	0.23	0.82
BMI, kg/m^2^, (M ± SD)	22.3 ± 4.1	23.2 ± 4.3	1.13	0.26
Sex (female), *n* (%)	111 (41.9)	8 (27.6)	2.22 *	0.14
APACHE II, score (M ± SD)	20.8 ± 8.2	19.9 ± 9.7	0.51	0.61
LOS hosp, days (M ± SD)	47.4 ± 31.1	42.1 ± 25.8	0.88	0.38
Hospital mortality, *n* (%)	37 (14.0)	14 (48.3)	21.47 *	<0.001
Re-intubation, *n* (%)	35 (13.2)	9 (31.0)	6.53 *	0.01
Tracheostomy, *n* (%)	171 (64.5)	17 (58.6)	0.40 *	0.53
Ventilator weaning, *n* (%)	223 (84.2)	13 (44.8)	25.52 *	<0.001

* = χ^2^ value. Quantitative variables: M ± SD; Qualitative variables: number and percentage of the group; VAE = ventilator-associated events; M ± SD = mean ± standard deviation; BMI = body mass index; APACHE = acute physiology and chronic health evaluation; LOS hosp = length of stay during hospitalization.

**Table 3:** Principal diagnostic reasons for tracheal intubation and clinical events occurring in the VAE group.

Principal Diagnostic Reason	*N* (%)
Total, *n* (%)	29 (100)
Stroke	6 (20.7)
Trauma	3 (10.3)
Pneumonia	7 (24.1)
Infection	3 (10.3)
Convulsion	3 (10.3)
Alveolar hemorrhage	2 (6.9)
Hemorrhagic shock	1 (3.4)
Others	4 (14.0)
Clinical events, *n* (%)	
Pneumonia	12 (41.4)
Pulmonary edema	10 (34.5)
Atelectasis	8 (27.6)
Shock	3 (10.3)
Others	3 (10.3)

VAE = ventilator-associated events. There was overlap in the clinical events considered as candidates.
